# Predictive value of motor-evoked potentials for upper limb functional outcomes in acute ischemic stroke

**DOI:** 10.1080/07853890.2025.2598930

**Published:** 2025-12-08

**Authors:** Cheng-Chang Yen, Hsin-Hung Chen, Yu-He Li, Ching-Huang Lin

**Affiliations:** ^a^ Division of Neurology, Department of Internal Medicine, Kaohsiung Veterans General Hospital, Kaohsiung, Taiwan; ^b^ Department of Medical Education and Research, Kaohsiung Veterans General Hospital, Kaohsiung, Taiwan; ^c^ Department of Laboratory Medicine, Zuoying Armed Forces General Hospital, Kaohsiung, Taiwan; ^d^ School of Medicine, College of Medicine, Sun Yat-sen University, Kaohsiung, Taiwan

**Keywords:** Acute ischemic stroke, Fugl-Meyer Assessment, functional recovery, motor-evoked potentials, modified Rankin Scale, prospective cohort study, transcranial magnetic stimulation, upper limb

## Abstract

**Background:**

Stroke remains a leading cause of acquired physical disability globally, with upper limb motor impairment significantly affecting independence and quality of life. Currently, objective neurophysiological biomarkers are not routinely incorporated into stroke care. This study aimed to investigate the predictive value of MEPs for upper limb outcomes in acute ischemic stroke.

**Patients and Methods:**

This prospective cohort study included 133 adults experiencing their first acute ischemic stroke. Within 10 days of onset, participants underwent Motor-evoked potential (MEP) testing via transcranial magnetic stimulation (TMS) and were categorized as MEP+ (with preserved responses) or MEP– (with absent responses). Outcome measures included the Fugl-Meyer Assessment (FMA), Barthel Index, and modified Rankin Scale (mRS), assessed at baseline and 90 days. Logistic regression models adjusted for age and baseline stroke severity were used to determine the independent predictive value of MEP status.

**Results:**

Among 133 patients (mean age 63.6 ± 12.2 years; 57.9% male), those with preserved MEPs (57.1%) demonstrated significantly better motor and functional outcomes at 90 days. MEP+ patients had higher FMA scores (61.0 ± 9.5 vs. 33.5 ± 25.5), Barthel Index scores (80.3 ± 25.0 vs. 57.7 ± 30.0), and greater odds of achieving minimal disability (mRS ≤ 1: 52.6% vs. 17.5%; all *p* < 0.001). Proportional recovery was also significantly higher in the MEP+ group for both the FMA and the Barthel Index (both *p* < 0.001).

**Conclusions:**

Early MEP assessment is a valuable prognostic tool for upper-limb stroke recovery, supporting its routine incorporation into clinical practice.

## Introduction

Stroke represents a major global health issue, severely impacting morbidity and mortality worldwide. It stands as the leading cause of acquired physical disability in adults and ranks as the second leading cause of death in middle-to-high-income countries, demonstrating the substantial burden it places on health systems and societies [1]. Over the last decade, the incidence rates for both ischemic and hemorrhagic stroke have risen, typically ranging from 85 to 94 cases per 100,000 individuals. Alarmingly, these rates escalate to 1151–1216 cases per 100,000 among those over 75 years old [2]. Low- and middle-income countries (LMICs) bear a disproportionate burden of stroke, accounting for approximately 85% of global stroke-related deaths and 87% of disability-adjusted life years (DALYs). Age-standardized stroke mortality rates in LMICs remain significantly higher than in high-income countries, reflecting disparities in primary prevention, access to acute care, and availability of post-stroke rehabilitation services [3]. In the United Kingdom alone, the economic burden of stroke is substantial, with annual costs related to stroke care and associated productivity losses amounting to approximately £8.9 billion, of which about 5% of total National Health Service expenses are dedicated to stroke care [4].

Upper limb motor impairment following stroke significantly affects functional independence and quality of life, with approximately 80% of stroke survivors experiencing initial upper limb weakness and only 20% achieving complete recovery. The heterogeneity in recovery outcomes necessitates the development of reliable prognostic tools to guide clinical decision-making and optimize rehabilitation strategies. While traditional clinical assessments provide valuable information, there is growing interest in neurophysiological biomarkers that can objectively evaluate the integrity of motor pathways and predict recovery potential. Motor recovery post-stroke is an essential focus of neurological research, with transcranial magnetic stimulation (TMS) playing a pivotal role in evaluating motor-evoked potentials (MEPs) and assessing corticospinal tract integrity [5]. The presence or absence of MEPs in the acute phase has emerged as one of the most robust predictors of motor motor outcomes after stroke, with numerous studies demonstrating its prognostic value across different stroke populations [6,7]. Stinear and colleagues have extensively investigated the predictive value of MEPs, developing the PREP (Predicting Recovery Potential) algorithm and its updated version, PREP2, which integrates MEP status with clinical and demographic information to provide individualized predictions of upper limb functional outcomes [8,9]. These prediction tools have shown superior accuracy compared to clinical assessments alone, with PREP2 achieving 75% accuracy in predicting upper limb function at 3 months post-stroke [9]. Multiple studies have reported strong associations between acute MEP presence and higher subsequent Fugl-Meyer scores (especially for distal upper limb function), underscoring the link between corticospinal integrity and motor outcome [10,11]. The FMA, as a comprehensive assessment tool for post-stroke motor function, has been extensively validated and is considered the gold standard for evaluating upper limb motor recovery [12,13]. Furthermore, acute MEP presence has been found to correlate with better activities of daily living, underscoring the functional relevance of corticospinal tract integrity [14]. The relationship between MEPs and stroke severity has also been established, with studies showing that corticospinal integrity (as indicated by MEP status) is associated with the clinical deficit level (NIHSS) [15,16]. More recently, studies have explored the integration of MEP status with other neurophysiological measures, such as somatosensory evoked potentials, to enhance prediction accuracy [17]. Despite the substantial evidence supporting the prognostic value of MEPs, several gaps remain in the literature. First, many studies have been limited by small sample sizes [6,7], which restricts their statistical power and generalizability to broader stroke populations. Second, there has been considerable variability in MEP assessment timing across studies, ranging from hyperacute evaluation within 72 h [18] to subacute assessment at 1–4 weeks post-stroke [19], making it difficult to establish standardized clinical protocols. Our study aims to address these gaps by investigating the predictive value of MEPs for upper limb motor outcomes in a large prospective cohort of patients with acute ischemic stroke. We hypothesized that the presence of MEPs in the acute phase (≤10 days post-stroke) will be significantly associated with better upper limb functional outcomes at 90 days, as measured by the FMA, Barthel Index, and modified Rankin Scale (mRS). Specifically, we aimed to determine whether early MEP status (≤ 10 days after stroke) can predict 90-day functional outcomes in stroke rehabilitation.

## Patients and methods

### Study design and participants

This prospective cohort study was conducted at Kaohsiung Veterans General Hospital between January 2018 and July 2024, enrolling adults who experienced their first-ever acute ischemic stroke. Inclusion criteria were: (1) ischemic stroke confirmed by neuroimaging, (2) clinically evident upper limb hemiparesis or hemiplegia, (3) admission within 10 days of symptom onset, (4) age ≥18 years, and (5) the ability to complete clinical assessments without cognitive interference. Exclusion criteria included a pre-stroke modified Rankin Scale score >1 and the presence of severe pre-existing conditions that could interfere with clinical evaluation or bias recovery outcomes. These conditions included sensory dysphasia, dementia, significant cognitive dysfunction, and severe psychiatric illnesses (e.g. schizophrenia or major depressive disorder with psychosis). Patients with contraindications to TMS were also excluded, such as those with metallic implants in the head (e.g. aneurysm clips, cochlear implants), cardiac pacemakers, a history of epilepsy, or pregnancy. Initially, 1382 patients were screened. After excluding those admitted beyond 10 days of stroke onset, those without clinically evident hemiparesis, and those with the above-mentioned exclusion criteria, 148 patients met all eligibility requirements. Of these, 133 completed the full study protocol and 90-day follow-up assessments. As illustrated in Supplementary Figure 1, the study was designed to evaluate the predictive value of motor-evoked potentials (MEPs) in forecasting upper limb functional outcomes following acute ischemic stroke. In addition, ischemic stroke subtypes were classified according to the TOAST (Trial of Org 10172 in Acute Stroke Treatment) criteria, which categorize strokes into five etiologies: large-artery atherosclerosis, cardioembolism, small-vessel occlusion, other determined causes, and undetermined causes. Classification was performed by attending stroke neurologists during hospitalization based on clinical evaluation, neuroimaging, vascular studies, and cardiac investigations. TOAST subtypes were used in descriptive analyses to explore potential associations with MEP status and functional outcomes.

### Ethical approval and patient consent

This study adheres to the Declaration of Helsinki. The institutional review board of Kaohsiung Veterans General Hospital approved the study protocol (IRB No. KSVGH21-CT5-46). Before being included in the study, all participants or their legal guardians provided written informed consent.

### Motor-Evoked Potentials Assessment

Within the first 10 days following stroke onset, all patients were subjected to TMS to assess the presence of MEPs in the upper limbs using a monophasic electromagnetic stimulator (Magstim 200 Mono Pulse, Magstim Company, Whitland, UK) with a 70 mm figure-of-eight coil, which generates a brief, high-intensity magnetic field that induces focal electrical currents in the underlying cortical tissue. The coil was positioned over the primary motor cortex (M1) contralateral to the affected upper limb, explicitly targeting the hand representation area. MEPs were recorded from the FDI muscle using surface electromyography with a Nicolet Viking IV system (Natus Medical, Pleasanton, CA). Ag/AgCl surface electrodes were placed in a belly-tendon montage, with the active electrode over the FDI muscle belly and the reference electrode over the metacarpophalangeal joint of the index finger. EMG signals were amplified (gain 1000), filtered (bandwidth 20–2000 Hz), and digitized at a sampling rate of 5 kHz. All recordings were performed with patients at rest, with instructions to remain relaxed and avoid voluntary muscle contraction. Single-pulse TMS was delivered with inter-stimulus intervals of 5–7 s to prevent cortical adaptation. Once the resting motor threshold (RMT) was determined, stimulation intensity was set at 120% of the RMT. MEP assessment used a binary presence/absence classification. The MEP with the shortest latency and greatest amplitude was taken for analysis. Participants were classified as MEP-positive if ≥2 of 5 trials elicited reproducible responses with a peak-to-peak amplitude of at least 50 μV at any intensity up to 100% maximum stimulator output (MSO). Participants were classified as MEP-negative if this positivity criterion was not met. To be completely clear for the reader, all TMS recordings were performed with the patient at rest, and no facilitation techniques (e.g. bilateral upper limb activation) were used. A patient was categorized as MEP-negative only if no MEPs could be elicited under these resting conditions, even at 100% MSO. All TMS assessments were performed by a trained neurophysiologist with more than 5 years of experience in clinical TMS. MEP traces were visually inspected for artifacts, and only artifact-free responses were included in the analysis. This binary classification approach has demonstrated superior prognostic accuracy compared to quantitative parameter analysis in previous stroke recovery studies [6,7].

### Functional assessments

Functional status in stroke patients within this study was assessed using four key tools: the NIH Stroke Scale (NIHSS), the Barthel Index, the Fugl-Meyer Assessment (FMA), and the modified Rankin Scale (mRS). The NIH Stroke Scale (NIHSS) measures the severity of stroke-induced neurological deficits. It evaluates several aspects, such as consciousness, gaze, visual field, facial palsy, motor strength, limb ataxia, sensory loss, language, and dysarthria, with scores ranging from 0 (no stroke symptoms) to 42 (most severe symptoms). The Barthel Index assesses the ability to perform ten basic activities of daily living, including feeding, moving from wheelchair to bed and returning, personal toilet, getting to and using the toilet, bathing, walking on a level surface, climbing stairs, dressing, controlling bowels, and controlling the bladder. Each activity is scored on a scale, contributing to a cumulative score ranging from 0 (completely dependent) to 100 (completely independent), offering a concrete measure of a patient’s functional independence. The FMA, specifically tailored for evaluating stroke recovery, focuses on motor function, balance, sensation, and joint functioning. For the upper extremities, it measures movement, strength, and coordination through a detailed examination using a 3-point ordinal scale (0–2) for each task, where 0 indicates total inability, 1 indicates partial ability, and 2 indicates full ability without difficulty. The maximum score for the upper extremities in this assessment is 66, representing the highest level of motor function achievable. Complementing the FMA, the mRS offers a broader assessment scale ranging from 0 (no symptoms) to 6 [2], which evaluates disability or dependence on daily activities. This scale helps to gauge the degree of disability and functional independence following a stroke. These assessments are conducted early, within 10 days post-stroke, to establish a baseline and are repeated at 90 days to monitor recovery and functional outcomes over time. This methodical approach enables a comprehensive evaluation of the relationship between early neurophysiological findings and long-term functional recovery, providing a thorough understanding of each patient’s recovery trajectory and the prognostic value of MEP assessment in stroke rehabilitation.

### Proportional recovery analysis

Proportional recovery, a concept first described by Prabhakaran et al. [20] and further developed by Stinear [21] and Winters et al. [22], represents the proportion of initially lost function that is regained during the recovery period. This measure has been extensively validated for upper limb motor function using the Fugl-Meyer Assessment, with studies consistently showing that most stroke patients recover approximately 70% of their initially lost motor function within the first 3 to 6 months post-stroke, regardless of the initial severity. For FMA scores, proportional recovery was calculated using the established formula: Proportional Recovery = (FMA_90_ - FMA_baseline)/(66 - FMA_baseline) × 100%, where 66 represents the maximum possible FMA upper limb score. This approach accounts for the ceiling effect by normalizing recovery relative to the potential for improvement, making it particularly valuable for comparing recovery across patients with different baseline severities. Regarding the Barthel Index’s proportional recovery, while this concept has been primarily established for motor function assessments, such as the FMA, recent studies have begun exploring its application to activities of daily living measures. We calculated Barthel Index proportional recovery using the formula: (BI_90_ - BI_baseline)/(100 - BI_baseline) × 100%, where 100 represents complete independence.

### Statistical analysis

The statistical analysis in this study on stroke recovery assessed differences in functional outcomes between groups identified by their Motor Evoked Potentials status (MEP+ and MEP-) using a comprehensive set of statistical tools. The initial analysis employed descriptive statistics to summarize patient demographics, clinical characteristics, and stroke specifics, presenting means, standard deviations for continuous variables, and frequencies for categorical variables. For comparative analysis, independent t-tests were utilized to evaluate differences in continuous variables, such as scores from the Fugl-Meyer Assessment, NIH Stroke Scale, and Barthel Index taken at the initial assessment. For categorical variables, the analytical approach depended on variable structure. Binary categorical variables such as sex, smoking status, and comorbidities were analyzed using 2 × 2 chi-square tests, with Fisher’s exact test applied when expected *p* < 0.05. For multi-categorical variables including stroke location and TOAST classification, overall chi-square tests of independence were performed to assess the association between MEP status and the complete distribution of categories, rather than conducting multiple pairwise 2 × 2 comparisons. This approach was chosen to avoid multiple comparison issues while maintaining statistical power to test the overall relationship between MEP status and categorical distributions. Outcome measures focused on the association between MEP status and functional outcomes, using logistic regression models to adjust for potential confounders, such as age and baseline stroke severity. These models specifically estimated the odds ratios for achieving favorable outcomes, defined as mRS ≤ 1 at 90 days, reflecting a favorable long-term functional outcome, and NIHSS ≤ 4 within 10 days post-stroke, as an indicator of mild neurological deficit in the acute phase. To establish a baseline for evaluating the prognostic utility of MEPs, we first assessed the independent predictive accuracy of various clinical scales for forecasting a favorable functional outcome at 90 days. Univariable logistic regression models were used to determine the predictive value of each clinical assessment, including the Fugl-Meyer Assessment (FMA), Barthel Index, and National Institutes of Health Stroke Scale (NIHSS), when treated as a continuous variable. The odds ratio (OR) for achieving a favorable outcome was calculated for each one-point increment in the respective scale. Next, to evaluate the discriminative ability of these scales, the Receiver Operating Characteristic (ROC) curve analysis was performed. The optimal cut-off point for each scale was determined by maximizing Youden’s index (Sensitivity + Specificity − 1). Using these data-driven cut-offs, we calculated the sensitivity, specificity, positive predictive value (PPV), and negative predictive value (NPV) for each scale in predicting a favorable 90-day outcome.

## Results

### Study implementation and patient characteristics

This prospective study included 133 patients experiencing their first ischemic stroke, with comprehensive demographic and clinical characteristics presented in Table 1. TMS for MEP assessment was performed at a mean of 6 ± 4 days post-stroke (range: 2–10 days), while clinical assessments, including NIHSS, Fugl-Meyer, and Barthel Index, were conducted at a mean of 7 ± 3 days (range: 4–10 days). Follow-up evaluations took place at 92 ± 5 days (range: 87–97 days). The timing of assessments did not differ significantly between the MEP+ and MEP − patients. Follow-up assessments occurred at 90 days post-stroke to evaluate recovery outcomes. Based on MEP assessment, 76 patients (57.1%) were classified as MEP+ and 57 patients (42.9%) as MEP-. Baseline upper limb motor function differed significantly between groups, with MEP+ patients demonstrating higher baseline FMA scores (48.8 ± 18.9) compared to MEP- patients (17.2 ± 19.1; *p* < 0.001), indicating that MEP+ patients had milder initial upper limb impairment.

**Table 1.** Clinical characteristics of all patients with stroke.

	Total (*n* = 133)	Range
Age (years), mean (*SD*)	63.6 (12.2)	(25–92)
Sex (male), *n* (%)	77 (57.9)	
Smoking, *n* (%)	49 (36.8)	
Drinking, *n* (%)	29 (21.8)	
BMI (kg/m^2^), mean (*SD*)	25.1 (4.4) / 132	(17.2–39.3)
Comorbidity		
Hypertension, *n* (%)	106 (79.7)	
Diabetic mellitus, *n* (%)	58 (43.6)	
Transient ischemic attack, *n* (%)	1 (0.7)	
Dyslipidemia, *n* (%)	107 (80.5)	
Atrial fibrillation, *n* (%)	19 (14.3)	
Myocardial infarction, *n* (%)	1 (0.7)	
Chronic renal disease, *n* (%)	3 (2.3)	
Chronic liver disease, *n* (%)	2 (1.5)	
COPD, *n* (%)	4 (3)	
Stroke Location		
Cortex, *n* (%)	23 (17.3)	
Subcortex, *n* (%)	78 (58.6)	
Brain stem, *n* (%)	31 (23.3)	
Cerebellum, *n* (%)	1 (0.7)	
Negative finding, *n* (%)	1 (0.7)	
TOAST classification		
Large artery, *n* (%)	45 (33.8)	
Cardioembolism, *n* (%)	25 (18.8)	
Small vessel, *n* (%)	21 (15.8)	
Cryptogenic, *n* (%)	31 (23.3)	
Undetermined, *n* (%)	11 (8.3)	
NIH Stroke Scale (NIHSS)	5.8 (4)	(0–18)
Barthel index	44.8 (28.1) /132	(0–100)
Fugl-Meyer assessment score, mean (*SD*)	35.5 (24.5)	(0–66)
MEP+, *n* = 76	48.8 (18.9)	(8–66)
MEP−, *n* = 57	17.2 (19.1)	(0–66)

Category variable: *n* (percentage), Continuous variable: mean (standard deviation) and range. BMI: body mass index; COPD: chronic obstructive pulmonary disease; NIH: National Institutes of Health; NIHSS: NIHSS test <10 days, Barthel index: Barthelindex test <10days, FMA: Fugl-Meyer assessment test <10 days; MEP: motor-evoked potential.

### Clinical characteristics by MEP status

Demographic characteristics were generally balanced between groups, with similar age distribution (MEP+ patients: mean age 64.1 ± 12.1 years vs MEP- patients: 63.0 ± 12.4 years) and sex distribution. As detailed in Table 2, MEP+ patients had significantly lower baseline NIHSS scores and higher Barthel Index scores compared to MEP- patients, suggesting milder initial stroke severity and better functional status in the MEP+ group. The distribution of stroke locations and TOAST classification patterns between groups is presented in Table 2.

**Table 2.** Clinical characteristics of all stroke patients with MEP+ and MEP.

	MEP+ (*n* = 76)	MEP− (*n* = 57)	*p*-Value, OR	*t*-test
Age (years), mean (*SD*)	64.1 (18.2)	63 (11.6		0.61
Sex (male), *n* (%)	43 (56.6)	34 (59.6)	0.86, 0.88 (0.44–1.77)	0.72
Smoking, *n* (%)	30 (39.5)	19 (33.3)	0.59, 1.3 (0.64–2.67)	0.47
Drinking, *n* (%)	18 (23.7)	11 (19.3)	0.67, 1.3 (0.56–3.02)	0.54
BMI (kg/m^2^), mean (*SD*)	25 (4.6) / 75	25.4 (4.2)		0.61
Comorbidity				
Hypertension, *n* (%)	58 (76.3)	48 (84.2)	0.29, 0.6 (0.25–1.47)	0.26
Diabetic mellitus, *n* (%)	31 (40.8)	27 (47.4)	0.48, 0.77 (0.38–1.53)	0.45
Dyslipidemia, *n* (%)	58 (77.6)	48 (84.2)	0.38, 0.65 (0.27–1.59)	0.34
Atrial fibrillation, *n* (%)	15 (19.7)	4 (0.7)	**0.046**, 3.26 (1.02–10.42)	**0.03**
Myocardial infarction, *n* (%)	1 (1.3)	0	1, 1.01 (0.99–1.04)	0.32
Chronic renal disease, *n* (%)	0	0	–	–
Chronic liver disease, *n* (%)	1 (1.3)	1 (1.7)	1, 0.75 (0.05–12.2)	0.84
COPD, *n* (%)	3 (3.9)	1 (1.7)	0.64, 2.3 (0.23–22.7)	0.44
Stroke Location				0.38
Cortex, *n* (%)	16 (21.1)	7 (12.3)	0.25, 1.91 (0.73 − 5)	***p* < 0.001**
Subcortex, *n* (%)	43 (56.6)	34 (59.6)	0.86, 0.88 (0.44–1.77)	0.72
Brain stem, *n* (%)	15 (19.7)	16 (28.1)	0.3, 0.63 (0.28–1.41)	0.27
Cerebellum, *n* (%)	1 (1.3)	0	1, 1.03 (0.99–1.04)	0.32
Negative finding, *n* (%)	1 (1.3)	0	1, 1.01 (0.99–1.04)	0.32
TOAST classification				**0.06**
Large artery, *n* (%)	29 (38.2)	16 (28.1)	0.27, 1.58 (0.75–3.31)	***p* < 0.001**
Cardioembolism, *n* (%)	19 (25)	6 (10.5)	**0.04**, 2.83 (1.05–7.65)	***p* < 0.001**
Small vessel, *n* (%)	9 (11.8)	12 (21.1)	0.16, 0.5 (0.2–1.29)	**0.02**
Cryptogenic, *n* (%)	11 (14.5)	20 (35.1)	**0.007**, 0.31 (0.14–7.3)	***p* < 0.001**
Undetermined, *n* (%)	8 (10.5)	3 (5.3)	0.35, 2.12 (0.54–8.37)	**0.004**
NIH Stroke Scale (NIHSS), mean (*SD*)	4 (2.9)	8.1 (4.2)		***p* < 0.001**
Barthel index, mean (*SD*)	54.3 (27.4) / 75	32.3 (23.8)		***p* < 0.001**
Initial FMA score, mean (*SD*)	48.8 (18.9)	17.2 (19.1)		**< 0.001**
mRS, mean (*SD*)	1.6 (1.2)	2.6 (1.1)		**< 0.001**

Category variable: *n* (percentage), Continuous variable: mean (standard deviation). Student’s *t*-tests for comparison of continuous variables and chi-square tests (or Fisher’s exact tests) for comparison of categorical variables between MEP + and MEP − patients.MEP: motor-evoked potentia; BMI: body mass index; COPD: chronic obstructive pulmonary disease; NIH: National Institutes of Health; NIHSS: NIHSS test <10 days; Barthel index: Barthel index test <10 days; FMA: Fugl-Meyer assessment test <10 days; mRS: Modified Rankin Scale. mRS at 90-day after stroke. The bold values highlight data points that show a statistically significant difference.

### Proportional recovery analysis

Proportional recovery analysis revealed significant differences between MEP groups, as presented in Table 3. For FMA scores, MEP+ patients achieved a mean proportional recovery of 78.6% ± 27.7%, indicating recovery of approximately three-quarters of their initially lost motor function. In contrast, MEP- patients achieved only 43.7% ± 37.3% proportional recovery (*p* < 0.001). Barthel Index proportional recovery showed a similar pattern, with MEP+ patients achieving 66.6% ± 34.7% compared to 43.4% ± 36.8% in MEP- patients (*p* < 0.001).

**Table 3.** Motor recovery after a stroke at different time points.

Post-stroke		MEP+	MEP-	
Barthel index score				*p*-Value
	≦10 days	54.3 (27.4)	32.3 (23.8)	
	90 days	80.3 (25)	57.7 (30)	*p* < 0.001
Proportional recovery (%)				
	≦10 days	0	0	
	90 days	66.6 (34.7)	43.4 (36.8)	*p* < 0.001
FMA score				*p*-Value
	≦10 days	48.8 (18.9)	17.2 (19.1)	
	90 days	61 (9.5)	33.5 (25.5)	< 0.001
Proportional recovery (%)				
	≦10 days	0	0	
	90 days	78.6 (27.7)	43.7 (37.3)	< 0.001

Mean (standard deviation).

MEP: motor-evoked potential; FMA: Fugl-Meyer assessment.

### Functional outcomes at 90 days

At 90-day follow-up, MEP+ patients demonstrated significantly superior functional outcomes across all measures, as shown in Table 3. MEP+ patients achieved higher FMA scores (61.0 ± 9.5 vs 33.5 ± 25.5, *p* < 0.001) and better Barthel Index scores (80.3 ± 25.0 vs 57.7 ± 30.0, *p* < 0.001) compared to MEP- patients. For functional independence, a significantly greater proportion of MEP+ patients achieved mRS ≤ 1 at 90 days compared to MEP- patients (52.6% vs 17.5%), with an adjusted odds ratio of 5.22 (95% CI: 2.31–11.83, *p* < 0.001), as detailed in Table 4.

**Table 4.** Prognostic value of MEPs for late and early functional outcomes after stroke.

	mRS ≤ 1 at 90 days	mRS > 1 at 90 days	Adjusted OR	95% CI(lower-upper)	*p*-Value
MEP+	40	36			
MEP−	10	47	5.22	2.31 − 11.83	< 0.001
	NIHSS < =4 at <10 day	NIHSS > 4 at <10 day			
MEP+	29	47			
MEP−	15	42	0.22^a^	0.1 − 0.47	< 0.001

^a^ Adjusted variables: age. OR: odds ratio; mRS: modified Rankin scale; NIHSS: NIH Stroke Scale; MEP: motor-evoked potential; CI: confidence interval.

### MEP status and concurrent neurological severity

We examined the association between MEP status and concurrent neurological severity within 10 days post-stroke, as measured by the NIHSS. This analysis revealed that MEP status was significantly associated with neurological severity, with fewer MEP– patients achieving favorable NIHSS scores (≤4) compared to MEP+ patients (15/57 vs. 29/76; adjusted OR = 0.22, 95% CI: 0.10–0.47, *p* < 0.001), as detailed in Table 4.

### Predictive performance of clinical scales for favorable functional outcome

To establish a clinical benchmark against which to compare the prognostic value of MEPs, we first evaluated the ability of standard clinical scales to predict a favorable functional outcome (mRS ≤ 1) at 90 days. In univariable logistic regression analyses, where scales were treated as continuous variables, several baseline assessments demonstrated significant predictive value (Supplementary Table 1). The baseline Fugl-Meyer Assessment (FMA) score emerged as a robust predictor, with each one-point increase being associated with a 7% higher odds of achieving a favorable outcome (OR = 1.07, 95% CI: 1.05–1.09, *p* < .001). The baseline Barthel Index score was also predictive, though to a lesser degree (OR = 1.03, 95% CI: 1.02–1.05, *p* < .001). Conversely, a higher baseline NIHSS score, indicating greater initial stroke severity, was associated with a reduced likelihood of a favorable outcome (OR = 0.72, 95% CI: 0.63–0.81, *p* < .001). Next, we assessed the discriminative performance of these scales using optimal cut-off points derived from ROC analysis (Table 5). The baseline FMA score demonstrated the strongest balanced performance, yielding a sensitivity of 82% and a specificity of 80% for predicting a favorable 90-day outcome. The baseline Barthel Index demonstrated a more modest performance, with a sensitivity of 68% and a specificity of 65%. The NIHSS, when dichotomized at its optimal cut-off, showed poor discriminative ability in this context.

**Table 5.** Predictive performance of clinical scales for a favorable 90-day functional outcome.

		Sensitivity	Specificity	PPV	NPV	*p*-Value
Barthel						
< =10 day		0.68 (0.56–0.80)	0.65 (0.55–0.76)	0.60 (0.48–0.72)	0.73 (0.63–0.84)	<0.001
90 day		0.54 (0.41–0.67)	0.83 (0.74–0.91)	0.70 (0.57–0.84)	0.70 (0.61–0.80)	<0.001
FMA score						
< =10 day		0.82 (0.73–0.92)	0.80 (0.71–0.89)	0.76 (0.65–0.86)	0.86 (0.78–0.94)	<0.001
90 day		0.81 (0.70–0.91)	0.75 (0.65–0.85)	0.71 (0.60–0.82)	0.84 (0.75–0.93)	<0.001
NIHSS	< =10 day	0.30 (0.18–0.42)	0.21 (0.12–0.30)	0.22 (0.13–0.31)	0.29 (0.17–0.40)	<0.001
mRS	90 day	0.40 (0.28–0.53)	0.20 (0.11–0.29)	0.27 (0.18–0.37)	0.31 (0.18–0.44)	<0.001

FMA: Fugl-Meyer assessment; NIHSS: NIH Stroke Scale; mRS: modified Rankin Scale; PPV: indicates predictive positive value; NPV: negative predictive value.

## Discussion

Early corticospinal integrity, indexed by transcranial magnetic stimulation motor‑evoked potentials, proved a powerful independent predictor of upper‑limb recovery in this prospective cohort of 133 first‑ever ischemic strokes. Patients with preserved MEPs (MEP+) not only presented with milder initial impairment but also achieved markedly superior 90‑day outcomes: mean Fugl‑Meyer Assessment (FMA) scores were almost doubled, Barthel Index gains were substantially larger, and the proportion reaching functional independence (mRS ≤ 1) was more than threefold higher than in patients without MEPs (MEP–). Adjusted odds of achieving mRS ≤ 1 remained ≈5‑fold greater in the MEP+ group after controlling for age and baseline NIHSS, confirming that the prognostic signal carried by MEPs is not simply a reflection of initial clinical severity. These findings extend earlier, smaller studies [6,7], including our prior 68 patient interim cohort from the same site [23], which is included within the present 133 patient sample, and confirm that MEPs retain predictive value when examined in a larger cohort of stroke patients.

The consistent advantage held by MEP+ patients reflects the underlying integrity of the corticospinal tract: intact pathways allow for more effective engagement of activity-dependent plasticity during rehabilitation, whereas severe tract damage constrains the potential for spontaneous restitution. This is consistent with network-based analyses showing that stronger sensorimotor connectivity, measured *via* the phase-lag index or directed-transfer function, correlates with higher FMA scores [24] and with studies demonstrating that delayed somatosensory evoked-potential latencies or disordered muscle synergies are associated with poorer motor performance [25]. In our cohort, MEP+ patients followed the classical proportional-recovery rule, regaining about 79 % of lost FMA points, close to the canonical 70 % predicted by biological recovery models [20,21]. By contrast, MEP– patients recovered only about 44 %, mirroring prior reports that absent MEPs frequently herald deviation from proportional recovery and suggest a need for compensatory rather than restorative strategies [26].

Although MEP+ patients entered rehabilitation with milder deficits, the prognostic influence of MEP status persisted after adjustment for baseline NIHSS and FMA, implying that neurophysiological information captures aspects of corticospinal integrity not evident in routine examination. This complements work underpinning the PREP and PREP2 algorithms, where integrating MEPs with simple bedside measures substantially improves individualized prediction accuracy [8,27]. MEP+ patients exhibited higher Barthel Index scores at 90 days, indicating greater independence in self-care and mobility. Although the proportional-recovery framework has been validated primarily for motor impairment, our exploratory proportional-Barthel analysis paralleled the motor findings, suggesting that similar biological limits may govern broader functional domains. These results align with large-scale Rasch analyses showing that Barthel and FIM-motor scores share a standard interval metric [28] and with reliability studies favorable outcomes confirming the responsiveness of Barthel’s scale in chronic stroke [29,30]. Superior modified Rankin Scale outcomes in the MEP+ group underscore the clinical relevance of early MEP testing, even though the mRS’s coarse granularity can mask domain‑specific gains [31,32].

Several significant limitations should be acknowledged. First, our findings represent group-level statistical associations rather than validated tools for individual patient prediction. While we demonstrate that MEP+ patients as a group have significantly better outcomes than MEP- patients, our study does not provide a practical, validated method for making accurate predictions for individual patients. Second, the sensitivity and specificity values we report indicate that MEP status alone is insufficient for precise individual prognostication, and substantial individual variation exists within both MEP+ and MEP- groups. A key limitation of our study is that we did not integrate MEP status with clinical variables into a comprehensive prediction tool, nor did we evaluate the performance of existing algorithms, such as PREP2, in our cohort. Third, we did not systematically evaluate whether combining MEP status with clinical scales would improve prediction accuracy beyond what either measure provides individually. Fourth, while proportional recovery has been well-established for FMA scores, its application to Barthel Index scores should be considered exploratory, as this concept has been primarily validated for assessments of motor function.

## Conclusions

**Figure 1.**
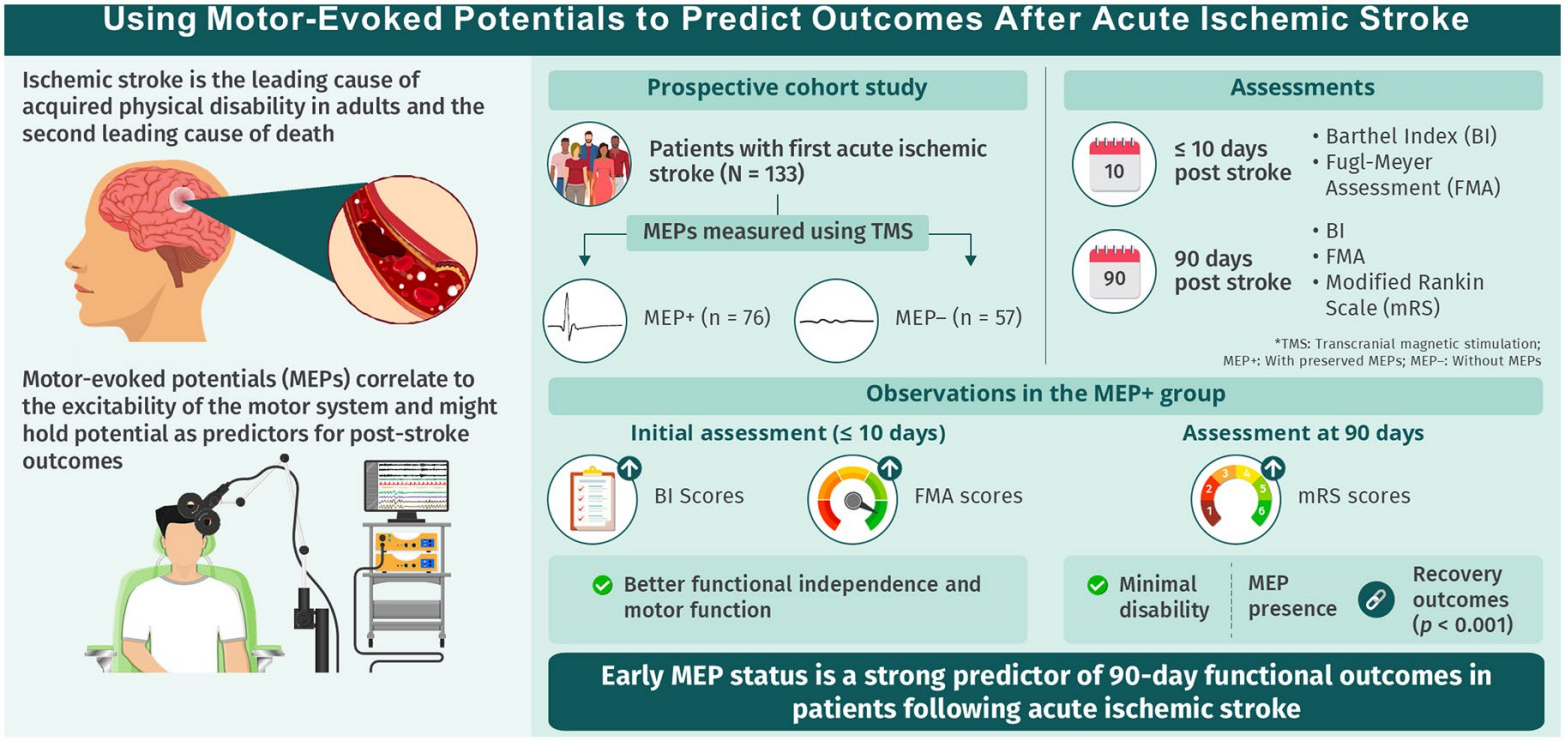
Using motor-evoked potentials to predict recovery after acute ischemic stroke. This prospective cohort study involved 133 patients who experienced their first acute ischemic stroke, aimed at predicting recovery using motor-evoked potentials (MEPs). MEPs were measured using transcranial magnetic stimulation (TMS), and patients were divided into two groups: those with preserved MEPs (MEP+, *n* = 76) and those without MEPs (MEP−, *n* = 57). Patients were assessed at two-time points: within 10 days post-stroke, where the Barthel Index [33] and Fugl-Meyer Assessment (FMA) were used to measure initial disability and motor function, and at 90 days post-stroke, where the BI, FMA, and Modified Rankin Scale (mRS) were utilized to evaluate outcomes. The findings show that patients in the MEP+ group exhibited better functional independence and motor function within the first 10 days, minimal disability, and improved outcomes at 90 days, with statistically significant differences in mRS scores (*p* < 0.001) compared to the MEP − group. These results indicate that early MEP status is a strong predictor 90-day functional outcomes in patients following acute ischemic stroke.

This study demonstrates that early MEP assessment provides valuable prognostic information for upper limb functional outcomes following acute ischemic stroke (Figure 1). MEP+ patients consistently achieved better outcomes across multiple functional domains, with proportional recovery rates consistent with established biological recovery patterns. In summary, our findings reinforce the prognostic value of acute MEP testing for upper limb outcomes after stroke. Future efforts should focus on the broader validation and implementation of individualized prediction tools that combine neurophysiological and clinical measures, building on the progress made to date.

## Supplementary Material

251028_Supplementary_Figure_1.docx

251028_Supplementary_Table_1.docx

## Data Availability

The datasets generated and/or analyzed during the current study are available from the corresponding author on reasonable request.

## References

[1] O’Donnell MJ, Chin SL, Rangarajan S, et al. Global and regional effects of potentially modifiable risk factors associated with acute stroke in 32 countries (INTERSTROKE): a case-control study. Lancet. 2016;388(10046):761–775. 27431356 10.1016/S0140-6736(16)30506-2

[2] GBD 2017 Causes of Death Collaborators. Global, regional, and national age-sex-specific mortality for 282 causes of death in 195 countries and territories, 1980-2017: a systematic analysis for the Global Burden of Disease Study 2017. Lancet. 2018;392(10159):1736–1788. 30496103 10.1016/S0140-6736(18)32203-7 PMC6227606

[3] Markus HS. The global burden of stroke. Int J Stroke. 2023;18(6):632–633. doi:10.1177/17474930231181677. 37376832

[4] Saka O, McGuire A, Wolfe C. Cost of stroke in the United Kingdom. Age Ageing. 2009;38(1):27–32. doi:10.1093/ageing/afn281. 19141506

[5] Groppa S, Oliviero A, Eisen A, et al. A practical guide to diagnostic transcranial magnetic stimulation: report of an IFCN committee. Clin Neurophysiol. 2012;123(5):858–882. doi:10.1016/j.clinph.2012.01.010. 22349304 PMC4890546

[6] Escudero JV, Sancho J, Bautista D, et al. Prognostic value of motor evoked potential obtained by transcranial magnetic brain stimulation in motor function recovery in patients with acute ischemic stroke. Stroke. 1998;29(9):1854–1859. doi:10.1161/01.str.29.9.1854. 9731608

[7] Rapisarda G, Bastings E, de Noordhout AM, et al. Can motor recovery in stroke patients be predicted by early transcranial magnetic stimulation? Stroke. 1996;27(12):2191–2196. doi:10.1161/01.str.27.12.2191. 8969779

[8] Stinear CM, Barber PA, Petoe M, et al. The PREP algorithm predicts potential for upper limb recovery after stroke. Brain. 2012;135(Pt 8):2527–2535. doi:10.1093/brain/aws146. 22689909

[9] Stinear CM, Byblow WD, Ackerley SJ, et al. PREP2: a biomarker-based algorithm for predicting upper limb function after stroke. Ann Clin Transl Neurol. 2017;4(11):811–820. doi:10.1002/acn3.488. 29159193 PMC5682112

[10] Stinear CM, Byblow WD, Ackerley SJ, et al. Proportional motor recovery after stroke: implications for trial design. Stroke. 2017;48(3):795–798. doi:10.1161/STROKEAHA.116.016020. 28143920

[11] Jo JY, Lee A, Kim MS, et al. Prediction of motor recovery using quantitative parameters of motor evoked potential in patients with stroke. Ann Rehabil Med. 2016;40(5):806–815. doi:10.5535/arm.2016.40.5.806. 27847710 PMC5108707

[12] Sullivan KJ, Tilson JK, Cen SY, et al. Fugl-Meyer assessment of sensorimotor function after stroke: standardized training procedure for clinical practice and clinical trials. Stroke. 2011;42(2):427–432. doi:10.1161/STROKEAHA.110.592766. 21164120

[13] Winstein CJ, Stein J, Arena R, et al. Guidelines for adult stroke rehabilitation and recovery: a guideline for healthcare professionals from the American Heart Association/American Stroke Association. Stroke. 2016;47(6):e98–e169. doi:10.1161/STR.0000000000000098. 27145936

[14] Lim KB, Kim JA. Activity of daily living and motor evoked potentials in the subacute stroke patients. Ann Rehabil Med. 2013;37(1):82–87. doi:10.5535/arm.2013.37.1.82. 23525518 PMC3604238

[15] Jung HY, Kim TH, Park JH. Relationship of national institute of health stroke scale and motor evoked potentials in subjects with stroke. Ann Rehabil Med. 2005;29(6):563–567.

[16] Pennisi G, Rapisarda G, Bella R, et al. Absence of response to early transcranial magnetic stimulation in ischemic stroke patients: prognostic value for hand motor recovery. Stroke. 1999;30(12):2666–2670. doi:10.1161/01.str.30.12.2666. 10582994

[17] Lee SY, Lim JY, Kang EK, et al. Prediction of good functional recovery after stroke based on combined motor and somatosensory evoked potential findings. J Rehabil Med. 2010;42(1):16–20. doi:10.2340/16501977-0475. 20111839

[18] Heald A, Bates D, Cartlidge NE, et al. Longitudinal study of central motor conduction time following stroke. 2. Central motor conduction measured within 72 h after stroke as a predictor of functional outcome at 12 months. Brain. 1993;116 (Pt 6)(6):1371–1385. doi:10.1093/brain/116.6.1371. 8293276

[19] Hendricks HT, Zwarts MJ, Plat EF, et al. Systematic review for the early prediction of motor and functional outcome after stroke by using motor-evoked potentials. Arch Phys Med Rehabil. 2002;83(9):1303–1308. doi:10.1053/apmr.2002.34284. 12235613

[20] Prabhakaran S, Zarahn E, Riley C, et al. Inter-individual variability in the capacity for motor recovery after ischemic stroke. Neurorehabil Neural Repair. 2008;22(1):64–71. doi:10.1177/1545968307305302. 17687024

[21] Stinear C. Prediction of recovery of motor function after stroke. Lancet Neurol. 2010;9(12):1228–1232. doi:10.1016/S1474-4422(10)70247-7. 21035399

[22] Winters C, van Wegen EE, Daffertshofer A, et al. Generalizability of the proportional recovery model for the upper extremity after an ischemic stroke. Neurorehabil Neural Repair. 2015;29(7):614–622. doi:10.1177/1545968314562115. 25505223

[23] Yen CC, Chen HH, Lee CH, et al. Predictive value of motor-evoked potentials for motor recovery in patients with hemiparesis secondary to acute ischemic stroke. Ann Med. 2023;55(1):2225144. doi:10.1080/07853890.2023.2225144. 37345693 PMC10288919

[24] Lee M, Kim YH, Lee SW. Motor impairment in stroke patients is associated with network properties during consecutive motor imagery. IEEE Trans Biomed Eng. 2022;69(8):2604–2615. doi:10.1109/TBME.2022.3151742. 35171761

[25] Williamson JN, Sikora WA, James SA, et al. Cortical reorganization of early somatosensory processing in hemiparetic stroke. J Clin Med. 2022;11(21):6449. doi:10.3390/jcm11216449. 36362680 PMC9654771

[26] Tscherpel C, Dern S, Hensel L, et al. Brain responsivity provides an individual readout for motor recovery after stroke. Brain. 2020;143(6):1873–1888. doi:10.1093/brain/awaa127. 32375172 PMC7296846

[27] Stinear CM, Barber PA, Smale PR, et al. Functional potential in chronic stroke patients depends on corticospinal tract integrity. Brain. 2007;130(Pt 1):170–180. doi:10.1093/brain/awl333. 17148468

[28] Prodinger B, O’Connor RJ, Stucki G, et al. Establishing score equivalence of the Functional Independence Measure motor scale and the Barthel Index, utilising the International Classification of Functioning, Disability and Health and Rasch measurement theory. J Rehabil Med. 2017;49(5):416–422. doi:10.2340/16501977-2225. 28471470

[29] Lee YC, Yu WH, Hsueh IP, et al. Test-retest reliability and responsiveness of the Barthel Index-based Supplementary Scales in patients with stroke. Eur J Phys Rehabil Med. 2017;53(5):710–718. doi:10.23736/S1973-9087.17.04454-9. 28178771

[30] Yang CM, Wang YC, Lee CH, et al. A comparison of test-retest reliability and random measurement error of the Barthel Index and modified Barthel Index in patients with chronic stroke. Disabil Rehabil. 2022;44(10):2099–2103. doi:10.1080/09638288.2020.1814429. 32903114

[31] Broderick JP, Adeoye O, Elm J. Evolution of the Modified Rankin Scale and its use in future stroke trials. Stroke. 2017;48(7):2007–2012. doi:10.1161/STROKEAHA.117.017866. 28626052 PMC5552200

[32] Erler KS, Wu R, DiCarlo JA, et al. Association of Modified Rankin Scale with recovery phenotypes in patients with upper extremity weakness after stroke. Neurology. 2022;98(18):e1877–e1885. doi:10.1212/WNL.0000000000200154. 35277444 PMC9109148

[33] Higgins JP, Altman DG, Gotzsche PC, et al. The Cochrane Collaboration’s tool for assessing risk of bias in randomised trials. BMJ. 2011;343:d5928. doi:10.1136/bmj.d5928. 22008217 PMC3196245

