# Supplementary material for: Predictive value of motor-evoked potentials for upper limb functional outcomes in acute ischemic stroke
Source: Ann Med. 2025 Dec 8;57(1):2598930. doi: 10.1080/07853890.2025.2598930 (PMC12687903; doi:10.1080/07853890.2025.2598930)
Supplement: 251028_Supplementary_Figure_1.docx [file IANN_A_2598930_SM6521.docx]

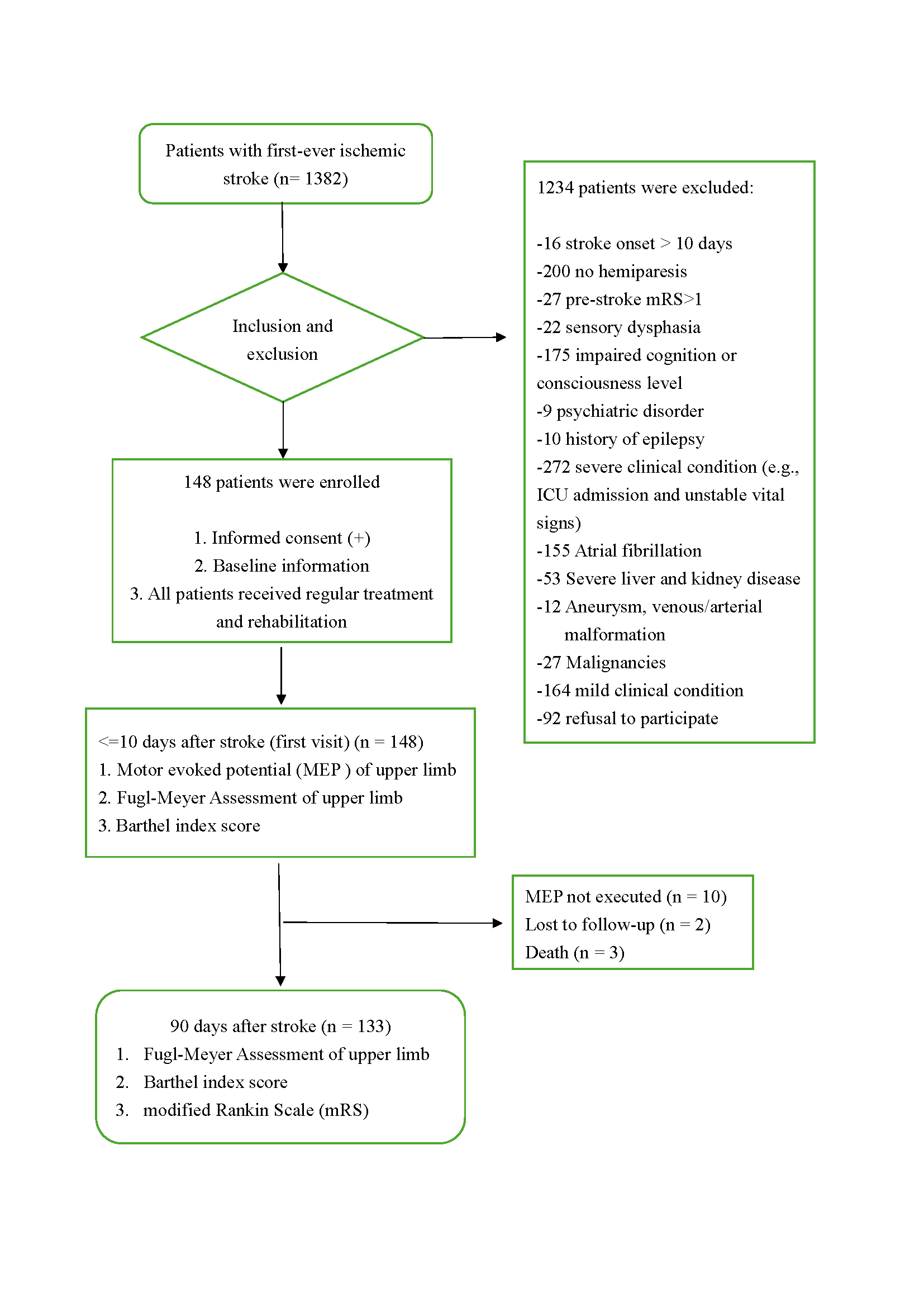


**Supplementary Figure 1. Study design and participant flow for evaluating the prognostic value of MEPs for upper limb functional outcomes in first-time acute ischemic stroke.**
