# Supplementary material for: Predictive value of motor-evoked potentials for upper limb functional outcomes in acute ischemic stroke
Source: Ann Med. 2025 Dec 8;57(1):2598930. doi: 10.1080/07853890.2025.2598930 (PMC12687903; doi:10.1080/07853890.2025.2598930)
Supplement: 251028_Supplementary_Table_1.docx [file IANN_A_2598930_SM6520.docx]

| **Supplementary Table 1. Logistic regression analysis of clinical scales predicting favorable functional outcomes at 90 days post-stroke** | | | | | |  |
| --- | --- | --- | --- | --- | --- | --- |
| Variables | β | S.E | Z | *P* | OR (95%CI) |  |
|  |  |  |  |  |  |  |
| Barthel <10 days | 0.03 | 0.01 | 4.23 | <.001 | 1.03 (1.02 ~ 1.05) |  |
| Barthel at 90 days | 0.03 | 0.01 | 4.16 | <.001 | 1.03 (1.02 ~ 1.04) |  |
| FMA <10 days | 0.07 | 0.01 | 6.34 | <.001 | 1.07 (1.05 ~ 1.09) |  |
| FMA at 90 days | 0.08 | 0.01 | 5.29 | <.001 | 1.08 (1.05 ~ 1.11) |  |
| NIHSS <10 days | -0.33 | 0.06 | -5.13 | <.001 | 0.72 (0.63 ~ 0.81) |  |
| mRS at 90 days | -0.75 | 0.17 | -4.36 | <.001 | 0.47 (0.34 ~ 0.66) |  |
| Favorable functional outcome was defined as a 90-day modified Rankin Scale score of ≤1. FMA: Fugl-Meyer assessment; NIHSS: NIH Stroke Scale; mRS: Modified Rankin Scale; β: Beta Coefficients; S.E.: Standard Error; OR: Odds Ratio, CI: Confidence Interval | | | | | |  |
